# Advances in hormone replacement therapy: making the menopause manageable

**DOI:** 10.1186/1472-6874-8-22

**Published:** 2008-11-27

**Authors:** Santiago Palacios

**Affiliations:** 1 Palacios Institute of Women's Health, Calle Antonio Acuña, 9, 28009, Madrid, España

## Abstract

The importance of the results of some large, randomized controlled trials (RCTs) on Hormone Replacement Therapy (HRT) has modified the risk/benefit perception of HRT. Recent literature review supports a different management.

The differences in age at initiation and the duration of HRT are key points. HRT appears to decrease coronary disease in younger women, near menopause; yet, in older women, HRT increases risk of a coronary event. Although HRT is a recognized method in the prevention and treatment of osteoporosis, it is not licensed for the prevention of osteoporosis as a first-line treatment. The effectiveness of low and ultra-low estrogen doses has been demonstrated for the treatment of vasomotor symptoms, genital atrophy and the prevention of bone loss, with fewer side-effects than the standard dose therapy. Further research, however, is needed to determine the effect both on fractures, as well as on cardiovascular and breast diseases. Newer progestins show effects that are remarkably different from those of other assays. The effectiveness of testosterone at improving both sexual desire and response in surgically and naturally postmenopausal women is shown by the testosterone patch.

The intention, dose and regimen of HRT need to be individualized, based on the principle of choosing the lowest appropriate dose in relation to the severity of symptoms and the time and menopause age.

## Introduction

By 2030, an estimated 47 million women will be undergoing menopause each year [1]. The loss of circulating estrogens that occurs during the menopausal transition manifests itself through a variety of symptoms (hot flushes, night sweats and vaginal atrophy). Approximately 75–80% of women experience menopausal symptoms, almost half of whom find the symptoms distressing, while 20–30% have severe symptoms [2,3].

For decades, estrogen, either alone or in combination with progestins, has been the therapy of choice for the relief of menopausal symptoms, as well as for the longer-term prevention of postmenopausal osteoporosis [3]. The results of two large studies on HRT (The Heart and Estrogen/progestin Replacement (HERS) Study and the Women's Health Initiative (WHI) Study), [4,5] [table 1], however, have modified the risk/benefit perception of HRT. This situation has been analysed by different scientific societies [6,7] which suggests its use in younger, recently menopausal women for symptomatic complaints (vasomotor and vaginal symptoms) and for primary prevention of postmenopausal osteoporosis. Moreover, the use of lower effective doses of hormones to achieve the desired objectives with the appropriate progestin is another issue. Finally, treatment with androgens for women with hypoactive sexual desire disorder should be considered.

**Table 1:** Key differences between the WHI and observational HRT studies

	**OBSERVATIONAL STUDIES**	**WHI**
AGE AT HRT INITIATION (MEAN)	52 yrs	63 yrs

TIME SINCE MENOPAUSE (MEAN)	1.5 yrs	12 yrs

VASOMOTOR SYMPTOMS	+	-

HRT FORMULATIONS	DIVERSES	CEE+MPA

DURATION OF HRT USE	LONG	SHORT

## Therapeutic window for starting HRT

The literature reveals that a number of studies on the effectiveness of HT have been carried out. Based on more than 40 observational studies of HRT and coronary heart disease (CHD), the summary relative risk for CHD was 40–50% lower among current or previous users of HT compared to those who never had used it (p < 0.001) [8]. In 2005 a Cochrane Review demonstrating an absence of benefit was published [9]. The latter data came from large prospective studies, such as HERS and WHI, and the results were consistent in that no benefit in secondary or primary prevention of cardiovascular disease (CVD) events was demonstrated.

Key differences between the WHI and observational studies of HT were the HT initiation and time since menopause [table 1]. The WHI Estrogen + Progestin (E+P) trial results according to time since menopause were the following: women <10 years since menopause, CHD Relative Risk (RR) = 0.89; women 10–19 years since menopause, RR = 1.22; and women 20 years and more since menopause, RR = 1.71 [10]. The WHI estrogen alone trial according to age at randomization concluded that the Myocardial Infarction (MI) or CHD death were the following: aged 50–59 years, RR = 0.63; 60–69 years, RR = 0.94; and 70–79 years, RR = 1.11. The absolute excess risk by age in the combined trials is completely different between the 50–59 year-old age group and the other age groups [table 2] [11]. Consequently, the differences in age at initiation and the duration of HRT use were sufficient to explain the discordance between the WHI trial and the observational studies. Furthermore, HRT appears to decrease coronary disease in younger women, near menopause; yet, in older women, HRT increases risk of a coronary event.

**Table 2:** Absolute risk (cases per 10.000 PYS) by age in the combined trials (E+P and E alone) of the WHI

**OUTCOME**	**AGE**		
CHD	50–59	60–69	70–79

TOTAL MORTALITY	-2	-1	+19

Modified Rossoux JAMA 2007	-10	-4	+16

## Bone loss prevention and treatment

At the time of menopause, estrogen deficiency initiates a rapid loss of Bone Mineral Density (BMD), a decrease of the micro-architectural deterioration leading to increased bone fragility and a higher risk of fracture. The results of the WHI study showed a significant reduction in all fractures in a population of patients who likely did not have significant fracture risk, based on the Body Mass Index (BMI), age and BMD results within the sub-group [5,12]. The data from the WHI study are the most robust non-vertebral fracture data extent. Another important aspect of the study to acknowledge is its quality, because of its sample size and the length of therapy. This study provides the largest database of any osteoporosis medication in randomized controlled trials (RCTs).

Although HRT in not licensed anymore for the prevention of osteoporosis as a first-line treatment, we think that HT seems to be the only proven effective option for the primary prevention of postmenopausal osteoporosis. It is a recognized method in the prevention and treatment of osteoporosis, which is confirmed by a meta-analysis of the efficacy of HRT in treating and preventing osteoporosis in postmenopausal women [13].

## The lower effective dose

The standard HRT doses, although effective, can be associated with adverse effects: breast cancer, venous thromboembolism and stroke being the most important. Several papers have indicated a dose dependency for HRT [14]. Therefore, it is logical to evaluate the effectiveness, the tolerability and the adverse effects of low doses of HRT [15].

The effectiveness of HRT in the relief of vasomotor symptoms in postmenopausal women is well established [16]. Several short-term studies have demonstrated a similar effectiveness for low doses compared to standard doses in order to alleviate hot flushes [17,18]. These promising initial results, suggesting the effectiveness of low doses, were confirmed by the HOPE study (Women's Health, Osteoporosis, Progestin, Estrogen study). This study evaluated conjugated equine estrogen (CEE) (0.3 or 0.45 mg/d) combined with medroxyprogesterone acetate MPA (1.5 or 2.5 mg/d). The study demonstrated that low doses were effective at diminishing the number and the intensity of hot flushes, and that those low doses seem to be as effective as the same CEE-MPA association at standard doses [19].

Other aspects deserve mention. Vasomotor symptoms appear in several short-term studies which demonstrate the effectiveness of low doses for the treatment of genital atrophy. Moreover, the HOPE study, with a larger series, also demonstrated that low doses are as effective as the standard ones for improving vaginal atrophy [19]. Still another aspect is the use of low doses as local treatment (estrogens for topical vulvo-vaginal administration) [20]. The adverse side effects of these topical treatments are less than 1/100, the most frequent being mucosa rash or very light allergic reactions with pruritus. Regarding CVD, there are few studies in relation to low doses. In a sample from the HOPE study, the impact of low doses on lipidic and carbohydrate metabolism were evaluated [21]. Another study concluded that low doses of CEE (0.3 mg/d) were as effective as the conventional ones (0.625 mg/d) at improving the lipid profile and the endothelial function [22].

The major drawback of all these studies are their short duration and the scant number of subjects included. Consequently, in order to assess the real effect of low doses on CVD, it is mandatory to design long-term trials which include a sufficient number of patients.

Bone density and fracture are both related to menopause. Data from the HOPE study suggest that low doses are effective at preventing the loss of bone density in spine and femur and at reducing bone turnover. The administration of calcium and vitamin D supplements facilitates the use of a lower dose of estrogen and guarantees an increase in bone mass in spine and femur similar to that observed using a standard dose [23]. At present there are no data correlating low doses and prevention of bone fractures. In earlier studies, drugs that seemed to reduce fracture incidence based on their effects on bone turnover have turned out to be really effective at reducing fractures in current studies [24]. The effect of low doses on bone turnover suggests a similar effect for the prevention of fractures [25]. There are results with a novel, continuous, ultra-low oral dose combined HRT with estradiol 0.5 mg that can alleviate subjective symptoms providing an effective protection against the postmenopausal decrease of BMD [26].

We can summarize that low dose and ultra-low dose therapies have shown to alleviate menopausal symptoms and have maintained or improved bone density with fewer side-effects than standard dose therapy. Nevertheless, further research is required to determine what effect the low and ultra-low dose therapy will have on fracture, cardiovascular and breast disease [27]. Consequently, an interesting option may be to begin HRT with low doses in order to minimize the side effects, and, if the administered dose eliminates or reduces the subjective symptoms, there is no reason to increase it.

## Appropriate progestin

For many years, progestins were considered as necessary additions to estrogen to protect the endometrium. However, while all exert progestagenic activity, they exhibit different patterns of binding at other steroid receptors and, consequently, display diverse biological activities [28] [table 3]. Indeed, different progestins may support or oppose the effects of estrogen, depending on the tissue, thereby supporting the concept that the clinical selection of progestins for HRT is critical in determining potential positive or detrimental effects [29]. Newer progestins, such as dydrogesterone drospirenone, show effects that are remarkably different from those of other assays; their actions might be particularly relevant to the cardiovascular system and the breast. Overall, it is not possible, given the profiles of different progestins, to make meaningful extrapolations from the results for one particular progestin to all progestins as a class nor indeed to all HRT agents because of their different progestin components [28]

**Table 3:** Comparison of the biological activities of progesterone and drospirenone with other progestogens (28)

**Progestogens**	**Biological activities**				
	**Progestogenic**	**Androgenic**	**Anti-androgenic**	**Anti-aldosterone**	**Glucocorticoid**

Progesterone	+	-	±	+	-

Drospirenone	+	-	+	+	-

Cyproterone acetate	+	-	+	-	±

Dienogest	+	-	+	-	-

Levonorgestrel	+	±	-	-	-

Medroxyprogesterone acetate	+	±	-	-	±

Norethisterone	+	±	-	-	-

Trimegestone	+	-	±	±	-

Norgestimate	+	±	-	-	-

Clinically relevant activity (+); activity not clinically relevant (±); no activity (-)

## Androgen hormonal therapy

The occurrence in some women of an androgen deficiency, inducing clinical symptoms and target tissue dysfunction, is plausible. Most of the controversy over this arises from the present difficulty of evaluating androgen activities in target tissues by using only serum measurements. In fact, the assays used to measure androgens have not been optimized to measure the low levels found in women. But there is evidence suggesting that testosterone might play an important role in different tissues and in modulating sexual response. The under-production of androgen in women, as may occur after bilateral oophorectomy, is associated with reduced sexual desire in some studies, but not in others [30].

Recently, the EMEA (European Agency for the Evaluation of Medical Products) approved the testosterone patch as a therapy for hypoactive sexual desire supported by clinical trials that show the effectiveness of testosterone at improving both sexual desire and response in surgically postmenopausal women. At present we have found five randomized placebo controlled studies (two phase II studies and three other phase III studies) with more than 2000 women studied [10]. In four cases, surgical menopause was carried out while in one case there was natural menopause. In all five cases, the efficacy and safety of a patch that released 300 μg of testosterone daily were analyzed [31-35]. All patients were treated with estrogens. The different results of the questionnaires used showed a significant increase in sexual desire and sexual response. In four out of five studies there was a significant reduction in the distress related to the problem of sexual dysfunction [31,33,34]. There are certain limitations to the five studies mentioned here. Only the safety data of the product have been evaluated in clinical trials of six months' duration; at present, there are data for 12 months as phase III has been prolonged into an open study. It will be necessary, however, to have a long term safety date.

## Conclusion

Despite the draw-back in hormone treatment for menopausal women during the last 3–5 years, there has been no argument about the efficacy and superiority of estrogen as the treatment of choice for menopausal symptoms. The recent randomized controlled studies have raised important issues that had not been dealt with before, such as the need to weigh benefits of therapy versus potential risks. Before treating with HRT, the indication, the balance of benefit-to-risk, the information given to the patient, and her acceptance of treatment must be valued. Finally, the dose and regimen of hormone therapy needs to be individualized based on the principle of choosing the lowest appropriate dose in relation to both severity of symptoms, as well as menopause age.

## Competing interests

Consultant, research grants and/or speaker for the following laboratories: Servier, Nycomed, Wyeth, Pfizer, Novartis, Amgen, Rovi, Bayer-Schering, Sanofi Pasteur-MSD, GSK, and Daiichi-Sankyo.

## Pre-publication history

The pre-publication history for this paper can be accessed here:


